# Ectopic ACTH secretion (EAS) associated to a well-differentiated peritoneal mesothelioma: case report

**DOI:** 10.1186/s12902-015-0031-4

**Published:** 2015-08-08

**Authors:** Carmen F. Mendoza, Patricia Ontiveros, Daniel X. Xibillé, Manuel H. Rivera

**Affiliations:** Endocrinology Department, Universidad Autónoma del Estado de Morelos and Hospital General de Cuernavaca, Iztaccihuatl Esq. Leñeros S/N, Los Volcanes, 62350 Cuernavaca, Morelos Mexico; Pathology Department, Hospital General de Cuernavaca, Morelos, Mexico; Universidad Autónoma del Estado de Morelos and Hospital General de Cuernavaca, Morelos, Mexico; Facultad de Medicina, Universidad Autónoma del Estado de Morelos, Cuernavaca, Morelos México

**Keywords:** Ectopic ACTH production, Hypercortisolism, Mesothelioma, Dexamethasone suppression test

## Abstract

**Background:**

The association between mesotheliomas and ectopic ACTH secretion has been rarely reported; we present the first case of ectopic ACTH secretion (EAS) associated with a well-differentiated peritoneal mesothelioma in whom the high dose dexamethasone suppression test (HDDST) results and plasmatic ACTH levels were similar to those found in Cushing’s disease (CD).

**Case presentation:**

A 43-year-old hispanic woman with a 20 year history of treatment resistant diabetes mellitus and arterial hypertension. She had a full moon face, a buffalo hump, increased volume in both supraclavicular regions, purple striae in her arms and abdomen, truncal obesity, polymenorrhea and umbilical hernia. A cortisol supression test with low dose dexamethasone (LDDST) with a result of 16.6 μg/dL and ACTH plasma levels were measured at 32.6 pg/mL. The high dose dexamethasone test suppression percentage was 84.8 % and magnetic resonance imaging (MRI) showed no evidence of pituitary alterations, computed tomography (CT) showed images suggestive of uterine fibroid and an intra-abdominal tumor that correlated with an umbilical hernia, which reinforcement after contrast. Surgery was performed, finding uterine fibroids and paracolic tumor implants as well as on the omentum, bladder, bowel, ovaries and appendix. Pathology reported a well-differentiated peritoneal mesothelioma with positive immunohistochemistry for ACTH.

**Conclusions:**

Although most cases of ectopic secretion of ACTH derive from rapidly-developing lung tumors, with very high plasma ACTH levels and cortisol suppression percentages with high doses of dexamethasone under 60 %, there is a small percentage of slow-developing, chronic tumors that are biochemically undistinguishable from Cushing’s disease. Following the expert recommendations regarding imaging techniques it is possible to identify the associated tumor in most cases.

## Background

Cushing’s syndrome is caused mainly by the use of glucocorticoids in supra-physiological doses. Endogenous Cushing’s syndrome is caused by an overproduction of cortisol by the adrenal glands, being of two types, ACTH dependent or independent; endogenous ACTH-dependent hypercortisolism is the most common (80-90 %) and it is caused, in about 90 % of cases, by a ACTH-producing pituitary adenoma [1, 2]. ACTH production by non-pituitary tumors represents approximately 10-15 % of ACTH-dependent Cushing’s syndrome. Reports concur that the tumors most commonly associated to ectopic ACTH secretion are bronchial carcinoid tumors, small-cell lung carcinoma, pulmonary adenocarcinoma, thymic carcinoid, medullary thyroid carcinoma and gastroenteropancreatic neuroendocrine tumors. Genitourinary tumors (prostate, bladder and ovarian endometrioid carcinoma) or metastatic neuroendocrine tumors of unidentified primary sites have been reported less frequently. In the St. Bartholomew series a mesothelioma was reported, diagnosed post-mortem, although histological confirmation was not obtained, and total hypophysectomy in this patient did not resolve the hypercortisolism [3–5]. There is an additional case reported in the literature, although it is a malignant pleural mesothelioma that clinically presented as coughing and pleuritic pain and was evidenced on thoracic x-rays [6]. Up to 20 % of cases with ectopic ACTH secretion derive from unidentified primary tumors [4, 7].

Peritoneal mesothelioma is a rare disease with a lineage derived from mesothelial cells. Its incidence is approximately one case per 1,000,000 persons and represents 20 to 30 % of all mesotheliomas. In general, the symptoms are not very specific and can be of two types, those related to the tumor mass, such as abdominal pain, constipation, nausea, vomiting, and ascites, or those that only present as ascites and abdominal distention. There have been reports of umbilical or inguinal hernias in some patients. There are two histopathological subtypes of peritoneal mesothelioma, the well-differentiated papillary mesothelioma (WDPM) and the benign multicystic mesothelioma. Both have a relatively indolent component, occurring mainly in women, are not related to asbestos and are treated by surgical resection. WDPM rarely suffers malignant transformation [8, 9]. We present the case of a patient with a 20-year history of Cushing’s syndrome, with a biochemical behavior similar to that of Cushing’s disease and whose histopathological diagnosis was a well-differentiated papillary mesothelioma.

## Case presentation

The patient is a 43 years old hispanic female with a history of left leg monoparesis as a complication of poliomyelitis. She was nubile, menarche occurred at age 14 and she initially had regular cycles. She was diagnosed with diabetes mellitus and hypertension at age 22, requiring treatment with 3 antihypertensive drugs in order to reach a stable control and used NPH insulin plus metformin for metabolic control; consequently, she has been diagnosed with non proliferative diabetic retinopathy and proteinuria. She underwent a diagnostic protocol for suspected Cushing’s disease 13 and 5 years prior to her visit to our center in two tertiary care centers, one of which performed an inferior petrosal sinus catheterization; the origin of the syndrome was never identified and the patient ceased going to subsequent appointments.

**Fig. 1:**
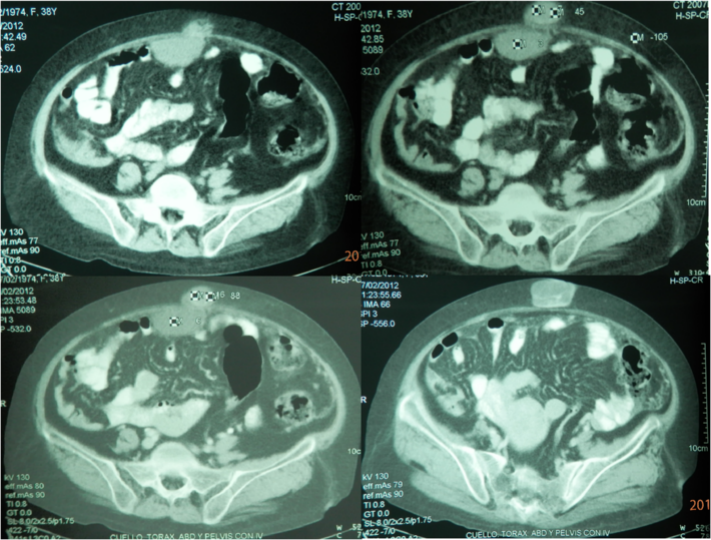
Abdominal computed tomography (CT) showed a bilobed image with protrusion through the umbilicus, with attenuation coefficients of 3, 7 and 45 HU; after administration of contrast enhancement they reached 6, 16 and 88 HU. Multiplanar reconstructions showed that its origin was intra-abdominal, adjacent to a loop of small intestine, and the possibility of a mesothelioma was considered

**Fig. 2:**
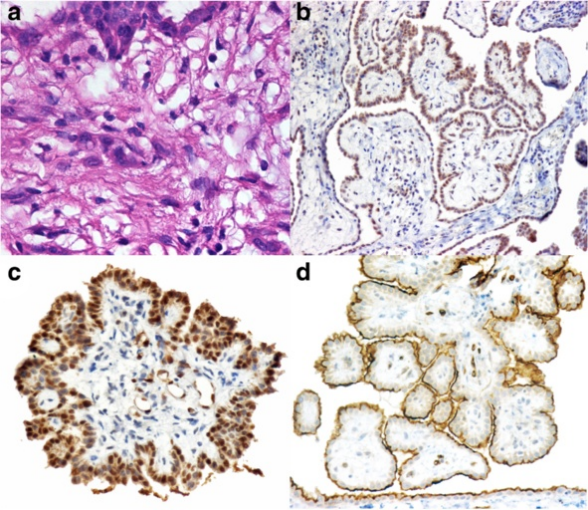
Immunohistochemistry. **(a)** A biopsy specimen showing a papillary pattern of the tumor and the absence of mitosis. (**b**)There is diffuse positivity for ACTH. Biopsy specimen positive to Calretinin (**c**) y thrombomodulin (**d**), supporting the diagnosis of mesothelioma

She was seen for the first time at our center 3 years prior; she came to the clinic complaining of polymenorrhea which had started 18 months prior, she had a full moon face, a buffalo hump, increased volume in both supraclavicular regions, purple striae in her arms and abdomen, truncal obesity, an umbilical hernia, decreased muscle mass of the pelvic limbs, uncontrolled glycemia, hypertension, albuminuria, a decreased glomerular filtration rate of 35 ml/min and osteoporosis. We performed the diagnostic protocol for Cushing’s syndrome again, as indicated in international guidelines; diagnostic tests showed the following data: urinary free cortisol: 186.5 μg/day, AM serum cortisol 21.83 μg/dL and midnight serum cortisol 16.09 μg/dL, serum cortisol post-1 mg dexamethasone (Low dose dexamethasone suppression test, LDDST): 16.6 μg/dL [all performed by chemiluminescent assay (CL), Accesses Immunoassay Systems], plasma ACTH: 32.6 pg/mL (CL), baseline AM serum cortisol: 17.8 μg/dL (CL), an overnight high-dose (8 mg PO) dexamethasone suppression test: 2.69 μg/dL (CL); TSH: 2.62 mIU/mL (0.50 to 5.0), free T4: 0.70 ng/dL (0.58 to 1.64) and free T3: 2.72 pg / mL (2.39 to 6.79); Total T4: 6.42 mg/dL (6.09 to 12.23) and Total T3: 0.88 ng/mL (0.87 to 1.78) [CL, Accesses immunoassay systems]. An MRI was performed in two different tertiary care centers due to the HDDST suppression percentage (84.8 %), both reporting a normal pituitary. Abdominal computed tomography (CT) showed a bilobed image with protrusion through the umbilicus, with attenuation coefficients of 3, 7 and 45 HU; after administration of contrast enhancement they reached 6, 16 and 88 HU, with a size of 68x60x76 mm. Multiplanar reconstructions showed that its origin was intra-abdominal, adjacent to a loop of small intestine, and the possibility of a mesothelioma was considered (Fig. 1). Images suggestive of uterine fibroids were also observed. Ultrasound imaging showed a normal liver, gallbladder and kidneys, with an enlarged uterus due to uterine fibroids and both ovaries showing no evidence of tumor or cystic lesions. The case was discussed with the General Surgery and Gynecology departments, and abdominal surgery was performed. The findings were: uterus with fibroids, abundant ascites and tumor implants in the omentum, bladder, bowel, ovaries and appendix. An intraoperative biopsy reported an ovarian carcinoma. A total hysterectomy, bilateral oophorectomy, omentectomy, appendectomy and umbilical hernioplasty was performed. The tumor implants were not completely removed due to the magnitude of their peritoneal extension. Finally, the pathology department, after analyzed the resected tissue, concluded that the patient had a well-differentiated papillary mesothelioma through staining with immunohistochemistry (Fig. 2).

Twenty four hours after surgery, her AM serum cortisol was measured (20.9 μg/dL). A month later the urinary free cortisol was 143.6 μg/day and serum cortisol post-1 mg dexamethasone was 19.1 μg/dL.

She is currently treated with ketoconazole 400 mg once a day, baseline insulin, calcium antagonists, statins, omega 3 fatty acids and conjugated estrogens. Despite not being cured, the patient does not want further treatment or any more invasive procedures. Her most recent laboratory results are: Plasma glucose 87 mg/dL, total cholesterol 145 mg/dL, triglycerides 172 mg/dL, HDL cholesterol 66.3 mg/dL, LDL cholesterol 73 mg/dL, creatinine 1.3 mg/dL, BUN 30 mg/dL and glycated hemoglobin 5.3 %.

## Discussion

It is known that non pituitary tumors have low cortisol suppression rates after high doses of dexamethasone, unlike those seen in pituitary adenomas; however, 10 % to 20 % of ectopic tumors can biochemically behave as Cushing’s disease. While several authors agree on the high sensitivity of the urinary free cortisol, late-night salivary cortisol and LDDST determination for the diagnosis of endogenous hypercortisolism, there is controversy about the usefulness of HDDST in locating an ACTH-producing tumor. Some authors, such as Aaron et al., suggest that the high dose dexamethasone test should no longer be used. They studied 68 patients with Cushing’s syndrome concluding that the accuracy of HDDST for differentiating between Cushing’s disease and ectopic ACTH secretion ranges between 70 % and 80 % [10]. However, in a study of 87 patients [11] in which 74.71 % were diagnosed with Cushing’s disease (CD), 8.05 % with EAS and 17.2 % with ACTH-independent Cushing’s syndrome, the mean serum cortisol suppression with 8 mgs of dexamethasone in the first group was 83.6 %, 52.5 % for the second and 17 % for the third group, showing statistically significant differences only between patients with Cushing’s disease and non-ACTH dependent Cushing’s syndrome, but not between the first group and those with ectopic ACTH secretion. This study concluded that the specificity of the cortisol suppression with high-dose dexamethasone test (HDDST) for the diagnosis of Cushing’s disease, with a percentage of suppression of more than 50 %, 60 % and 79 %, is 75 %, 88 % and 100 % respectively [11]. In the series published by Vilar et al., 74 cases of patients with endogenous hypercortisolism were analyzed; seven had EAS and of these 28.6 % had a suppression of >50 % of serum cortisol levels with HDDST, and the only patient who had a rate of suppression of >80 % had Cushing’s disease, so these authors suggest that a percentage of suppression of >80 % in HDDST should be achieved to distinguish between Cushing’s disease and EAS [12]. In another series published by Isidiri [3], Doi [4], Kakade [5] and Ejaz [13], percentages of serum cortisol suppression using HDDST in patients with EAS were no greater than 50 %, with few exceptions. The gold standard to distinguish between Cushing’s disease and ectopic ACTH secretion, in patients with discordant results (suppression of cortisol post-HDDST >60 % and normal IRM), is a bilateral inferior petrosal sinus sampling (BIPSS), a ratio of central to peripheral ACTH of more than 2 under baseline conditions or over 3 after CRH stimulation, which is consistent with Cushing’s disease. If the diagnosis of Cushing’s disease is excluded after BIPSS, the next step is to perform a CT scan or MRI of the neck, thorax, abdomen and pelvis to identify the non-pituitary ACTH-producing tumor [14, 15]; however, it has been described that in up to 20 % of patients the site of ectopic ACTH secretion cannot be identified. Because most neuroendocrine tumors express receptors for somatostatin, some case reports have mentioned the use of somatostatin receptor scintigraphy (SRS) in order to identify the origin of the ectopic ACTH secretion; nonetheless, Tabarin et al., reviewed 20 cases of ectopic ACTH secretion published in the literature finding that in 18 of the 20 cases, the tumor was visible using more conventional methods (CT scans and MRI), as well as SRS. The same authors present a series of 12 patients with ectopic ACTH secretion who underwent serial CT or MRI scanning and SRS, with the latter resulting in findings in only four of the patients, but in two of them the tumor detected both by SRS as by conventional imaging, was not the origin of the ectopic ACTH secretion, with the primary tumor remaining unknown. Another patient dies before the tumor, identified using SRS, could be resected and, in the last case, the image seen as abnormal in SRS turned out to be a carcinoid metastasis from an unknown primary site, unidentified by conventional imaging [16]. In the series by Doi, et al., 50 % of tumors causing ectopic ACTH secretion were identified through CT or MRI, and 11 of the 16 cases presented underwent somatostatin receptor scintigraphy (SRS), four of which were positive (36 %), with only one (11.1 %) positive to [^18^ F] fluorodeoxyglucose-positron emission tomography (FDG-PET) [4]. On the other hand, in the series of patients published by Ejaz, et al., CT or MRI identified the origin of the ectopic ACTH secretion in 67.5 %, failing to find it in four patients in spite of performing CT, MRI, octreoscan and FDG-PET [13]. In another series of 19 patients, the usefulness of SRS with In 111 octreotide and Ga 68 DOTATATE PET-CT was evaluated, being positive in only 7 of these patients, 4 of which had undergone a previous CT that had shown the sites of abnormal uptake, and another in which the MRI had also shown an abdominal tumor; one more who had already undergone resection of a mediastinal mass and lymph nodes without showing clinical improvement underwent SRS which showed lymph node and bone metastasis. The last one had a pulmonary tumor, diagnosed with Ga 68 DOTATATE and had previously undergone a CT that failed to reveal the tumor. The authors suggest that performing SPECT or SPECT/TC imaging would improve the sensitivity of diagnosis with the planar images of SRS and propose that the images with Ga 68 peptide may offer better results in localizing ectopic ACTH-producing tumors than those peformed with In 111 octreotide [17]. There are, to date, several case reports in which imaging with Ga 68 DOTATATE have identified the tumor secreting ectopic ACTH [18–20] but it must be remembered that case reports involve a selection bias per se in evaluating the sensitivity of a diagnostic tool. Most authors currently coincide that once the ectopic secretion of ACTH is confirmed through BIPSS, imaging techniques such as CT or MRI should be preferentially employed in an initial stage as most tumors will be shown by their use. In the case here presented, abdominal CT evidenced an intra-abdominal tumor and the possibility of a mesothelioma was considered as the most likely, leading to an exploratory laparotomy in the patient, without performing other imaging tests, as SRS or PET/CT are difficult to access in most of our country.

Finally, the histological diagnosis of peritoneal mesothelioma was performed on the basis of a positive staining for keratin markers such as calretinin and thrombomodulin, as well as negative staining using markers for adenocarcinoma such as carcinoembrionic antigen (CEA), CA-125 and CK 5/6. Calretinin is expressed in normal and neoplastic mesothelial cells, with some authors reporting positive staining for this protein in 80 % of mesotheliomas with a specificity close to 100 % while, for thrombomodulin, some studies have reported a positivity of 80 % in mesotheliomas and only 15 % in adenocarcinomas. The sensitivity also depends on whether mono or poyclonal antibodies are employed, as well as the tissue fixation in large specimens [21–23].

A fact that stands out in the case we present is that the levels of plasma ACTH were on the lower limit of the normal range. Although most patients with ectopic ACTH secretion have plasma levels that are considerably greater than normal, there are also reports of cases with normal or mildly elevated ACTH; these cases generally have a gradual progression and are associated to poorly aggressive carcinoid tumors. Neary, et al, presented a series of 12 patients with ACTH-producing neuroendocrine thymic tumors where 2 cases had normal plasma ACTH in spite of having considerably high levels of free urinary cortisol [24]. On the other hand, in a series of 90 patients with EAS published by the National Institutes of Health, 30 % had normal baseline plasma ACTH, one of them with an ACTH of 12.7 pg/mL [25], leading to the conclusion that levels of plasma ACTH per se do not rule out a suspicion of ectopic secretion of ACTH.

## Conclusions

The differential diagnosis between Cushing’s disease and ectopic secretion of ACTH is a challenge. The case presented here is a rare example of those reaching a suppression percentage with HDDST which was similar to Cushing’s disease. Most authors have found, and statistics confirm this, that with suppression percentages over 80 %, the probability that the origin of Cushing’s syndrome is a tumor is very high. When MRI is repeatedly normal, as in the case here presented, the best diagnostic test is an inferior petrosal sinus catheterization. This test was performed in our patient 13 years prior in a tertiary care center and, although we were not able to review the results, the patient did not undergo pituitary surgery and was only followed in the outpatient clinic, leading us to assume that the result reflected ectopic secretion without localizing the origin of the secretion at that time because the clinical course of the patient had been insidious, presenting new symptoms and signs more than 20 years after the onset of the first clinical manifestations, which differs from most non-pituitary ACTH-producing tumors, whose development is faster and more aggressive.

## Consent

Written informed consent was obtained from the patient for publication of this case report and any accompanying images. A copy of the written consent is available for review by the Editor of this journal.

## Abbreviations

EAS: Ectopic ACTH secretion
CD: Cushing’s disease
HDDST: High dose dexamethasone suppression test
LDDST: Low dose dexamethasone suppression test
MRI: Magnetic resonance imaging
CL: Chemiluminescent assay
CT: Computed tomography
WDPM: Well-differentiated papillary mesothelioma
SRS: Somatostatin receptor scintigraphy
FDG PET: [^18^ F] fluorodeoxyglucose-positron emission tomography

## References

[1] Pivonello R, De Martino MC, De Leo M, Lombardi G, Colao A (2008). Cushing’s syndrome. Endocrinol Metab Clin N Am.

[2] Bertagna X, Guignat L, Groussin L, Bertherat J (2009). Cushing’s disease. Best Pract Res Clin Endocrinol Metab.

[3] Isidori AM, Kaltsas GA, Pozza C, Frajese V, Newell-Price J, Reznek RH, Jenkins PJ, Monson JP, Grossman AB, Besser GM (2006). The ectopic adrenocorticotropin syndrome: clinical features, diagnosis, management, and long term follow-up. J Clin Endocrinol Metab.

[4] Doi M, Sugiyama T, Izumiyama H, Yoshimoto T, Hirata Y (2010). Clinical features and management of ectopic ACTH syndrome at a single institute in Japan. Endocrin Journal.

[5] Kakade HR, Kasaliwal R, Jagtap VS, Bukan A, Budyal SR, Khare S, Lila AR, Bandgar T, Menon PS, Shah NS (2013). Ectopic ACTH-secreting syndrome: a single center experience. Endocr Pract.

[6] Lee JM, Pou K, Sadow PM, Chen H, Hu B, Hewison M, Adams JS, Sugarbaker DJ, Fisher ND (2008). Vitamin D-mediated hypercalcemia and Cushing syndrome as manifestations of malignant pleural mesothelioma. Endocr Pract.

[7] Ray DW (2006). Ectopic adrenocorticotropin syndrome: diagnosis and treatment. Curr Opin Endocrinol Diabetes.

[8] Sugarbaker PH, Acherman YI, Gonzalez-Moreno S, Ortega-Perez G, Stuart OA, Marchettini P, Yoo D (2002). Diagnosis and treatment of peritoneal mesothelioma: The Washington Cancer Institute experience. Semin Oncol.

[9] Kindler Hedy Lee. Peritoneal mesothelioma: the site of origin matters. Am Soc Clin Oncol Educ Book*.* 2013:182-8. doi: 10.1200/EdBook_AM.2013.33.182 10.14694/EdBook_AM.2013.33.182 23714495

[10] Aron DC, Raff H, Findling JW (1997). Effectiveness *versus* efficacy: the limited value in clinical practice of high dose dexamethasone suppression testing in the differential diagnosis of adrenocorticotropin-dependent Cushing’s syndrome. J Clin Endocrinol Metab.

[11] Günes M, Celik O, Kadioglu P (2013). Reliability of the diagnostic tests for Cushing’s syndrome performed in a tertiary referral center. Pituitary.

[12] Vilar L, Freitas MC, Naves LA, Canadas V, Albuquerque JL, Botelho CA, Egito CS, Arruda MJ, Silva LM, Arahata CM, Agra R, Lima LH, Azevedo M, Casulari LA (2008). The role of non-invasive dynamic tests in the diagnosis of Cushing’s syndrome. J Endocrinol Invest.

[13] Ejaz S, Vassilopoulou-Sellin R, Busaidy NL, Hu MI, Waguespack SG, Jimenez C, Ying AK, Cabanilas M, Abbara M, Habra MA (2011). Cushing syndrome secondary to ectopic adrenocorticotropic hormone secretion. Cancer.

[14] Boscaro M, Arnaldi G (2009). Approach to the patient with possible Cushing’s syndrome. J Clin Endocrinol Metab.

[15] Nieman LK, Biller BMK, Findling JW, Newell-Price J, Savage MO, Stewart PM, Montori VM (2008). The diagnosis of Cushing’s syndrome: an endocrine society clinical practice guideline. J Clin Endocrinol Metab.

[16] Tabarin A, Valli N, Chanson P, Bachelot Y, Rohmer V, Bex-Bachellerie V, Catargi B, Roger P, Laurent F (1999). Usefulness of somatostatin receptor scintigraphy in patients with occult ectopic adrenocorticotropin syndrome. J Clin Endocrinol Metab.

[17] Gözde Özkan Z, Kuyumcu S, Balköse D, Özkan B, Aksakal N, Yılmaz E, Şanlı Y, Türkmen C, Aral F, Adalet I (2013). The value of somatostatin receptor imaging with In-111 octreotide and/or Ga-68 DOTATATE in localizing ectopic ACTH producing tumors. Mol Imaging and Radionucl Ther.

[18] Venkitaraman B, Karunanithi S, Kumar A, Bal C, Ammini AC, Kumar R (2014). ^68^Ga-DOTATOC PET-CT in the localization of source of ectopic ACTH in patients with ectopic ACTH-dependent Cushing’s syndrome. Clin Imaging.

[19] Willhauck MJ, Pöpperl G, Rachinger W, Giese A, Auernhammer CJ, Spitzweg C (2012). An unusual case of ectopic ACTH syndrome. Exp Clin Endocrinol Diabetes.

[20] Singer J, Werner F, Koch CA, Bartels M, Aigner T, Lincke T, Fasshauer M, Pascke R (2010). Ectopic Cushing’s syndrome caused by a well differentiated ACTH-secreting neuroendocrine carcinoma of the ileum. Exp Clin Endocrinol Diabetes.

[21] Stolnicu S, Quiñonez E, Boros M, Molnar C, Dulcey I, Nogales F. Case report: papillary mesothelioma of the peritoneum with foamy cell lining. Diagn Pathol. 2013;8:1–4. 10.1186/1746-1596-8-162 PMC3853708 24066870

[22] Barnetson RJ, Burnett RA, Downie I, Harper CM, Roberts F (2006). Immunohistochemical analysis of peritoneal mesothelioma and primary and secondary serous carcinoma of the peritoneum. Am J Clin Pathol.

[23] Attanoos RL, Webb R, Dojcinov SD, Gibbs AR (2001). Malignant epithelioid mesothelioma: anti-mesothelial marker expression correlates with histological pattern. Histopathology.

[24] Neary NM, López-Chávez A, Abel BS, Boyce AM, Schaub N, Kwong K, Stratakis CA, Moran CA, Giaccone G, Nieman LK (2012). Neuroendocrine ACTH-Producing tumor of the tymus-experience with 12 patients over 25 years. Clin Endocrinol Metab.

[25] Ilias I, Torpy DJ, Pacak K, Mullen N, Wesley RA, Nieman LK (2005). Cushing’s syndrome due to ectopic corticotropin secretion: twenty years’ experience at the National Institutes of Health. J Clin Endocrinol Metab.

