# Erratum to: Ectopic ACTH secretion associated to a well-differentiated peritoneal mesothelioma

**DOI:** 10.1186/s12902-015-0046-x

**Published:** 2015-09-14

**Authors:** Carmen F. Mendoza, Patricia Ontiveros, Daniel X. Xibillé, Heriberto Manuel Rivera

**Affiliations:** Endocrinology Department, Universidad Autónoma del Estado de Morelos and Hospital General de Cuernavaca, Iztaccihuatl Esq. Leñeros S/N, Los Volcanes, Cuernavaca, 62350 Morelos Mexico; Pathology Department, Hospital General de Cuernavaca, Morelos, Mexico; Universidad Autónoma del Estado de Morelos and Hospital General de Cuernavaca, Morelos, Mexico; Facultad de Medicina, Universidad Autónoma del Estado de Morelos, Cuernavaca, Morelos México

## Erratum to: BMC Endocrine Disorders doi: 10.1186/s12902-015-0031-4

Unfortunately the publication the original version of this article in *BMC Endocrine Disorders* [1] contained an error. The funding of the paper was not mentioned in the original version. However, the published paper was funded by the Fondo Mixto-Consejo Nacional de Ciencia y Tecnología and Morelos Government [Grant MOR-2013-C01-224236] and the Programa de Mejoramiento del Profesorado [Grant 103.5/13/8913 PTC-300] (to H.M.R.). Additionally, the name of author Heriberto Manuel Rivera was incorrectly spelled as Manuel H. Rivera, Author Heriberto Manuel Rivera is the corresponding author of the paper instead of author Carmen F. Mendoza.
